# An Investigator-Initiated Phase 2 Study of Nivolumab Plus Low-Dose Ipilimumab as First-Line Therapy for Microsatellite Instability—High Advanced Gastric or Esophagogastric Junction Cancer (NO LIMIT, WJOG13320G/CA209-7W7)

**DOI:** 10.3390/cancers13040805

**Published:** 2021-02-15

**Authors:** Hisato Kawakami, Shuichi Hironaka, Taito Esaki, Kazuaki Chayama, Masahiro Tsuda, Naotoshi Sugimoto, Shigenori Kadowaki, Akitaka Makiyama, Nozomu Machida, Hidekazu Hirano, Kenro Hirata, Hiroki Hara, Hiroshi Yabusaki, Yoshito Komatsu, Kei Muro

**Affiliations:** 1 Department of Medical Oncology, Faculty of Medicine, Kindai University, Osaka 589-8511, Japan; 2 Department of Medical Oncology and Hematology, Faculty of Medicine, Oita University, Oita 879-5593, Japan; shironaka@oita-u.ac.jp; 3 Department of Gastrointestinal and Medical Oncology, National Hospital Organization Kyushu Cancer Center, Fukuoka 811-1395, Japan; taitoesaki@gmail.com; 4 Department of Gastroenterology and Metabolism, Graduate School of Biomedical and Health Sciences, Hiroshima University, Hiroshima 734-8551, Japan; chayama@mba.ocn.ne.jp; 5 Department of Gastroenterological Oncology, Hyogo Cancer Center, Hyogo 673-8558, Japan; b4hkgsntgm7@hp.pref.hyogo.jp; 6 Department of Medical Oncology, Osaka International Cancer Institute, Osaka 541-8567, Japan; sugimoto-na2@mc.pref.osaka.jp; 7 Department of Clinical Oncology, Aichi Cancer Center Hospital, Aichi 464-8681, Japan; skadowaki@aichi-cc.jp (S.K.); kmuro@aichi-cc.jp (K.M.); 8 Cancer Center, Gifu University Hospital, Gifu 501-1194, Japan; makiyama20@hotmail.com; 9 Department of Gastroenterology, Kanagawa Cancer Center, Kanagawa 241-8515, Japan; n-machida@kcch.jp; 10 Gastrointestinal Medical Oncology Division, National Cancer Center Hospital, Tokyo 104-0045, Japan; hihirano@ncc.go.jp; 11 Division of Gastroenterology and Hepatology, Department of Internal Medicine, School of Medicine, Keio University, Tokyo 160-8582, Japan; kenro916@gmail.com; 12 Department of Gastroenterology, Saitama Cancer Center, Saitama 362-0806, Japan; hirhara@cancer-c.pref.saitama.jp; 13 Department of Gastroenterological Surgery, Niigata Cancer Center Hospital, Niigata 951-8566, Japan; yabu@niigata-cc.jp; 14 Department of Cancer Chemotherapy, Hokkaido University Hospital Cancer Center, Hokkaido 060-8648, Japan; ykomatsu@ac.cyberhome.ne.jp

**Keywords:** gastric cancer, microsatellite instability, nivolumab, ipilimumab

## Abstract

**Simple Summary:**

Microsatellite instability-high (MSI-H) is an established biomarker for response to immune checkpoint inhibitors (ICIs). ICIs are not usually administered in the first-line setting for MSI-H tumors including gastric cancer (GC), although such tumors tend to be less responsive to cytotoxic chemotherapy compared with microsatellite-stable tumors. On the basis of evidence suggesting that nivolumab plus low-dose ipilimumab can improve survival in MSI-H colorectal cancer, we plan to investigate the efficacy and safety of this regimen for MSI-H GC, which accounts for ~5% of all GC cases. The NO LIMIT study (WJOG13320G/CA209-7W7) is an investigator-initiated, single-arm, open-label, 14-center phase 2 trial of nivolumab plus low-dose ipilimumab for MSI-H GC in the first-line setting. Its primary objective is to determine the overall response rate for the study treatment as assessed by blinded independent central review. The planned number of subjects is 28.

**Abstract:**

Nivolumab (NIVO) plus low-dose ipilimumab (IPI) has shown a promising survival benefit in first-line treatment of microsatellite instability-high (MSI-H) colorectal cancer. We hypothesized that this regimen might also be beneficial for MSI-H gastric cancer (GC), which accounts for ~5% of all GC cases. NO LIMIT (WJOG13320G/CA209-7W7) is an investigator-initiated, single-arm, open-label, 14-center phase 2 trial of NIVO plus low-dose IPI for MSI-H GC in the first-line setting. Eligibility criteria include unresectable advanced, recurrent, or metastatic gastric or esophagogastric junction cancer with a histologically confirmed diagnosis of adenocarcinoma; confirmed MSI-H status with the MSI-IVD Kit (FALCO); no prior systemic anticancer therapy; an Eastern Cooperative Oncology Group performance status of 0 or 1; and a measurable lesion per RECIST 1.1. The primary objective of the study is to determine the overall response rate (ORR) for the NIVO+IPI regimen as assessed by blinded independent central review. Secondary end points include progression-free survival, overall survival, duration of response, safety, tolerability, and biomarkers. The number of patients was set at 28 on the basis of the threshold and expected ORR values of 35 and 65%, respectively, with a one-sided alpha error of 0.025 and power of 0.80. Subjects will receive treatment with nivolumab (240 mg) biweekly in combination with ipilimumab (1 mg/kg) every 6 weeks. The results of this study should clarify the therapeutic potential of NIVO+IPI for MSI-H GC in the first-line setting. Trial registration: JapicCTI-205400.

## 1. Introduction

Gastric cancer (GC) remains one of the most common and deadly cancers worldwide, especially among older males. GLOBOCAN 2018 data show that stomach cancer is the fifth most common neoplasm and the third most deadly cancer, with an estimated 783,000 deaths in 2018 [1]. GC patients often present with unresectable or metastatic disease at diagnosis, with cytotoxic chemotherapy having the potential to prolong survival and improve quality of life (QoL) in such individuals. However, such chemotherapy can deliver only a moderately longer duration of disease control and survival, with its efficacy having reached a plateau. There is thus a strong need for new and more effective therapies for this malignancy.

Cancer immunotherapy with immune checkpoint inhibitors (ICIs) has recently shown clinical activity and the ability to confer a survival benefit either as monotherapy or in combination with other types of immunotherapy or conventional chemotherapy in multiple types of cancer. In the case of GC, monotherapy with nivolumab, an antibody to programmed cell death-1 (PD-1), conferred a significantly longer overall survival (OS) compared with placebo in the third- or later-line setting in the ATTRACTION-2 study [2], resulting in nivolumab becoming the standard of care in this setting in Japan. However, the proportion of patients who benefit from ICIs is smaller for GC than for other types of solid tumor, highlighting the importance of biomarker identification for selection of individuals most likely to benefit from such treatment.

Efforts are underway to identify biomarkers that can predict the response of ICIs in GC. Studies have suggested several potential candidates with various levels of evidence including microsatellite instability-high (MSI-H), PD-L1, and Epstein–Barr virus positivity in tumor [3,4]. Among them, MSI-H has been an established biomarker for ICIs across tumor types. The prevalence of MSI-H was found to be ~5% in Japanese patients with metastatic or recurrent GC [5]. The clinicopathologic profile of MSI-H tumors includes a local and systemic antitumoral immune response. The initial control of MSI-H tumors by immune surveillance provides a strong rationale for ICI treatment, given its mechanism of action based on abrogation of immune tolerance. Indeed, pembrolizumab, another antibody to PD-1, has shown efficacy for MSI-H tumors across tumor types [6,7,8] and has thus been approved in Japan for MSI-H, unresectable or metastatic solid tumors that have progressed after previous treatment. Among such tumors, colorectal cancer (CRC) has been studied most closely. The KEYNOTE-177 study recently showed that pembrolizumab monotherapy in the first-line setting conferred a superior progression-free survival (PFS) [9] as well as a better QoL [10] compared with chemotherapy for patients with MSI-H CRC (Table 1), resulting in pembrolizumab monotherapy becoming the standard first-line treatment for such patients in the United States. The KEYNOTE-062 phase 3 study evaluated pembrolizumab with or without chemotherapy versus chemotherapy alone in the first-line setting for GC, demonstrating noninferiority of pembrolizumab monotherapy relative to chemotherapy in patients with a programmed cell death-ligand 1 (PD-L1) combined positive score (CPS) of ≥1 [11]. Consistent with the findings of KEYNOTE-177, pembrolizumab monotherapy tended to confer a better outcome compared with chemotherapy in the MSI-H subgroup of these patients in KEYNOTE-062 [12] (Table 2), although the number of such cases was limited (*n* = 14 for pembrolizumab monotherapy versus *n* = 19 for chemotherapy).

Nivolumab in combination with ipilimumab, an antibody to cytotoxic T lymphocyte-associated protein-4 (CTLA-4), is another treatment option for MSI-H CRC. A multicohort, nonrandomized phase 2 study (CheckMate-142) revealed substantial efficacy for nivolumab (3 mg/kg) plus ipilimumab (1 mg/kg) every 3 weeks (four doses) followed by nivolumab (3 mg/kg) every 2 weeks, with a trend toward a higher overall response rate (ORR) and longer survival even in the second-line setting [13] compared with nivolumab monotherapy. This combination of drugs (nivolumab plus ipilimumab (NIVO+IPI)) was thus approved for the second-line treatment of MSI-H CRC in the United States as well as in Japan. Preliminary data for the first-line cohort in the CheckMate-142 study also revealed highly promising efficacy for nivolumab (3 mg/kg) every 2 weeks plus ipilimumab (1 mg/kg) every 6 weeks [14] (Table 3). Evidence thus suggests that NIVO+IPI has the potential to become a standard treatment for MSI-H CRC in the first-line setting, with this potential awaiting confirmation by the results of an ongoing phase 3 study (CheckMate-8HW) [15], in which the efficacy and safety of NIVO+IPI are being compared with those of nivolumab monotherapy and conventional chemotherapy.

Previous studies have indicated that MSI tumors have a better prognosis [16,17] than microsatellite-stable (MSS) tumors do, whereas the efficacy of cytotoxic chemotherapy for MSI-H cancers is not necessarily greater than that for MSS tumors [16,17]. 5-Fluorouracil-based adjuvant chemotherapy is not recommended for MSI-H stage II CRC because of a lack of efficacy [16,17]. For MSI-H GC, data suggest that neither perioperative (MAGIC trial) [18] nor adjuvant (CLASSIC trial) [19] chemotherapy improves survival when added to surgery, consistent with the results of preclinical studies showing chemoresistance in MSI-H tumors [20,21]. The use of adjuvant chemotherapy for MSI-H early-stage GC is thus controversial [22,23]. In the metastatic setting, evidence suggests that both MSI-H CRC and MSI-H GC have a poor prognosis after treatment with cytotoxic chemotherapy [24,25]. These findings are thus suggestive of a limited positive or even detrimental effect of cytotoxic chemotherapy for MSI-H tumors.

Given that the efficacy of ICIs for MSI-H cancer is evident across tumor types, the findings for NIVO+IPI in MSI-H CRC might be reproducible in MSI-H GC. We therefore plan to conduct an open-label, multicenter phase 2 trial (NO LIMIT) of nivolumab plus low-dose ipilimumab for MSI-H gastric tumors.

## 2. Study Design and Methods

### 2.1. Objectives

The primary end point of the study is ORR as assessed by blinded independent central review (BICR) for NIVO+IPI in subjects with MSI-H GC. Secondary end points include ORR by investigator assessment; disease control rate (DCR), defined as the proportion of complete responses (CRs), partial responses (PRs), and stable disease (SD) and determined by BICR and investigator assessment; PFS by BICR and investigator assessment; OS; the duration of response (DoR), determined by BICR and investigator assessment and defined as the time from the date of first documented response (CR or PR) to the date of first disease progression (per RECIST 1.1) or death due to any cause, whichever occurs first; time to response, determined by BICR and investigator assessment and defined as the time from enrollment to the date of first documented response (CR or PR, as per RECIST 1.1); overall safety and tolerability of treatment; and concordance rate for MSI-H between the MSI-IVD Kit (FALCO) and other assays as well as exploration of potential biomarkers associated with clinical efficacy (ORR, PFS, or OS) or with the incidence of adverse events of NIVO+IPI treatment with the use of archival tumor tissue and prospectively collected blood samples.

### 2.2. Study Design

The study is a single-arm phase 2 trial of NIVO+IPI in adult (≥20 years of age) male and female patients with unresectable advanced, recurrent, or metastatic MSI-H GC. A total of 28 subjects is planned. The planned enrollment period is 2 years (1 November 2020 to 31 October 2022), and the planned follow-up period is 2 years beginning after the day the last patient is enrolled. The planned study period is thus 4 years (1 November 2020 to 31 October 2024). Patients will receive nivolumab at a dose of 240 mg as a 30 min (±10 min) intravenous infusion on day 1 of each 2-week treatment cycle as well as ipilimumab at a dose of 1 mg/kg as a 30 min (±10 min) infusion every three cycles (that is, every 6 weeks). Treatment with NIVO+IPI will be administered for up to 24 months in the absence of disease progression or unacceptable toxicity. Treatment with NIVO+IPI can be reinitiated as per the initial schedule after disease progression and administered for up to 1 additional year. Even in this latter case, however, the maximum administration period for the study treatment after its initial onset is 24 months.

### 2.3. Study Population

Subjects must meet all eligibility criteria of the protocol. The key inclusion and exclusion criteria are shown in Table 4.

### 2.4. Study Assessments

Subjects will be assessed for response by computed tomography or magnetic resonance imaging as per Response Evaluation Criteria in Solid Tumors (RECIST) version 1.1. Assessments will be performed at baseline (within 28 days prior to enrollment), every 3 treatment cycles for the first 24 cycles, and then every 6 cycles, regardless of study drug administration, until disease progression (unless treatment beyond progression is permitted), subsequent anticancer therapy, loss to follow-up, or withdrawal of consent, whichever comes first. Subjects who discontinue the study treatment for reasons other than disease progression and who continue in the follow-up phase of the study will continue to have tumor assessments every 6 weeks (or every 12 weeks after 1 year from the first study drug administration). All images obtained for tumor assessment in all enrolled subjects will be submitted to BICR for determination of response (according to RECIST 1.1) and study end points. Adverse events and laboratory values will be graded according to the National Cancer Institute (NCI) Common Terminology Criteria for Adverse Events (CTCAE) version 5.

### 2.5. Sample Size Calculation and Statistical Analysis

The sample size is based on the primary objective: determination of ORR for patients with MSI-H GC treated with NIVO+IPI. The threshold value (35%) is based on the response rate for chemotherapy in MSI-H patients in the KEYNOTE-062 trial [12], that for nivolumab monotherapy in patients with MSI-H CRC in the second-line cohort of the CheckMate-142 trial [13], and that for pembrolizumab monotherapy in patients with MSI-H non-CRC tumors in the KEYNOTE-158 trial [6]. The expected value (65%) is based on the response rate for NIVO+IPI in patients with MSI-H CRC in the first-line setting of CheckMate-142 [14] (Table 3). With an alpha error of 0.025 (one-sided) and power (1−β) of 0.80, we calculated the minimum number of patients to be 26. Taking into account patient dropout, we set the required number of study subjects at 28.

### 2.6. Biomarker Analysis

Tumor tissue and blood will be collected prior to enrollment. Samples for serum and buffy coat will also be collected on day 1 of cycle 4, and samples for serum will be collected at the time of progression or suspected progression.

Tumor specimens collected before enrollment will be analyzed for immune cell populations and expression of selected tumor markers. Tumor tissue will be examined with the Oncomine Immune Response Research Assay (OIRRA) to detect expression of selected immune-related genes. OIRRA is an RNA-based next-generation sequencing (NGS) assay that allows characterization of transcript abundance for ~400 genes relevant to oncology and immune therapy response research. RNA and DNA from tumor samples will also be analyzed with the Oncomine Comprehensive Assay (OCA) Plus, which is a pan-cancer, multi-biomarker NGS-based assay that allows comprehensive genomic profiling of key targeted therapy and immunotherapy biomarkers such as tumor mutation load and microsatellite status. This assay covers >500 distinct genes and supports simultaneous analysis of both DNA and RNA in one workflow.

Treatment with NIVO+IPI will also be investigated in relation to T cell receptor (TCR) and B cell receptor (BCR) repertoire analysis of peripheral blood specimens collected from all subjects prior to enrollment or during treatment. The data will be evaluated for associations with response, survival, or safety. The TCR repertoire will be analyzed by interrogating the TCR β-chain locus with the Oncomine TCR Beta-LR Assay, as reported recently [26]. The BCR repertoire will be analyzed by interrogating the BCR heavy-chain (immunoglobulin heavy chain, IGH) locus with the Oncomine BCR IGH-LR Assay.

### 2.7. Study Organization and Conflict of Interest

This trial is supported by Bristol-Myers Squibb but will be conducted as an investigator-initiated clinical study. The West Japan Oncology Group (WJOG) controls the conflicts of interest for researchers involved in the study and persons supporting the study.

### 2.8. MSI-H Screening Program

To identify 28 eligible patients with MSI-H GC, which accounts for only ~5% of all GC cases, we have already started another nationwide observational study (WJOG13320GPS/CA209-7W6; trial registration, UMIN000040366) to screen 1000 chemotherapy-naive Japanese patients with recurrent or metastatic GC with the use of the MSI-IVD Kit (FALCO). This kit is able to detect MSI-H status with DNA isolated only from tumor tissue (without the need for a corresponding blood sample) on the basis of multiplex PCR (polymerase chain reaction) fragment analysis with five mononucleotide repeat markers that were designed by Promega and which have a low susceptibility to genetic polymorphisms [27]. The MSI-IVD Kit has obtained companion diagnostic approval from the Ministry of Health, Labor, and Welfare of Japan.

The primary objective of the WJOG13320GPS/CA209-7W6 study is to determine the frequency of MSI-H for unresectable advanced, recurrent, or metastatic GC not previously treated with chemotherapy. Secondary objectives include characterization of pathological and clinical background factors, which has not been investigated as extensively as MSI-H CRC, time to test for MSI (turnaround time), and MSI test success rate in patients with MSI-H GC. MSI-H cases identified by the WJOG13320GPS/CA209-7W6 study will be eligible for enrollment in the NO LIMIT study. The concordance of MSI status determined with the MSI-IVD Kit versus that determined by other assays including immunohistochemistry and NGS will be evaluated in this study, given that misdiagnosis of MSI status potentially leads to de novo invalidity of ICI treatment.

## 3. Discussion

Given that both CTLA-4 and PD-1 have cell-intrinsic regulatory activity, simultaneous blockade of both molecules can lead to functional convergence through enhancement of T-cell activity with several mechanisms [28]. In Japan, NIVO+IPI has been approved for treatment of metastatic melanoma and renal cell carcinoma, as well as more recently for that of metastatic MSI-H CRC in the second-line setting on the basis of the results of the CheckMate-142 study [13]. This drug combination has consistently shown higher efficacy than nivolumab monotherapy, although a higher incidence of immune-related adverse events (irAEs) including colitis and hepatitis has been a major concern. A variety of NIVO+IPI regimens, with different doses and schedules, has been investigated (Table 5).

Data suggest that a higher dose intensity of ipilimumab is more likely to be associated with a higher incidence of adverse events that can lead to treatment discontinuation. In the case of GC, different dosing schedules of NIV PI were evaluated in the CheckMate-032 study (Table 3), with a high dose of ipilimumab (3 mg/kg) in combination with nivolumab at 1 mg/kg (NIVO1+IPI3) showing a manageable safety profile and moderate efficacy [32], and the subsequent CheckMate-649 phase 3 study therefore adopted NIVO1+IPI3 for comparison with chemotherapy with or without nivolumab. However, new patient enrollment in the NIVO1+IPI3 arm of this trial was discontinued on the basis of a recommendation from the data-monitoring committee [34]. On the other hand, favorable safety as well as efficacy have been achieved for other tumor types in studies with nivolumab at 3 mg/kg every 2 weeks plus low-dose ipilimumab (1 mg/kg) every 6 weeks, as in the CheckMate-227 phase 3 trial for NSCLC [33] and in the first-line cohort of the CheckMate-142 study for MSI-H CRC [14]. We therefore adopted this dosing regimen for NIVO+IPI in the NO LIMIT study, in line with the aforementioned CheckMate-8HW study of MSI-H CRC [14]. To explore biomarkers potentially associated with safety of NIVO+IPI in the NO LIMIT study, we plan to investigate the relation of severe irAEs to TCR haplotypes detected by the TCR-long read (TCR-LR) assay [35]. If it proves possible to predict irAEs on the basis of TCR analysis with the use of a blood sample collected at screening, then NIVO+IPI will become even more widely accepted.

In MSI-H CRC, the survival and QoL benefits of first-line pembrolizumab monotherapy relative to conventional chemotherapy were demonstrated in the KEYNOTE-177 study [9,10]. The important message here is that MSI-H CRC should be treated differently from MSS CRC, and we hypothesized that this distinction may also be applicable to other tumor types. However, in the KEYNOTE-177 study, the proportion of patients experiencing disease progression in the pembrolizumab monotherapy group was higher than that in the conventional chemotherapy group (29.4% versus 12.3%) [9]. Other than misdiagnosis of MSI status, a possible reason for this finding is that MSI-H tumors show highly up-regulated expression of multiple immune checkpoint proteins including CTLA-4 [36], which may also explain, in part, the better efficacy of NIVO+IPI compared with nivolumab alone in the CheckMate-142 study [13]. In the NO LIMIT study, the factors associated with efficacy of or resistance to NIVO+IPI will be comprehensively captured and analyzed with the OCA Plus and OIRRA assays, which should provide further insight into the immune mechanisms relevant to MSI-H tumors and possible optimal treatment options for nonresponders.

A global phase 3 trial (CheckMate-649) recently found that the combination of nivolumab and chemotherapy (FOLFOX or capecitabine plus oxaliplatin) conferred a significant survival benefit in previously untreated patients with advanced GC in a manner dependent on PD-L1 expression level [37]. Furthermore, an Asian phase 3 trial (ATTRACTION-4) showed that nivolumab plus chemotherapy (oxaliplatin in combination with capecitabine or S-1) achieved an unprecedented median PFS for GC of >10 months in this setting [38]. These results suggest that the combination of nivolumab and chemotherapy may become a standard first-line treatment for GC. However, further analysis with longer follow-up of the efficacy of nivolumab plus chemotherapy for MSI-H GC is warranted, given the potential chemoresistance of MSI-H tumors and preclinical data addressing the association between repeated cycles of 5-fluorouracil and impairment of antitumor immune function [39]. NIVO+IPI, a non-cytotoxic chemotherapeutic regimen, will likely have advantages in this regard, and the results of the NO LIMIT study may provide a novel treatment option for patients with MSI-H GC, who are less likely to benefit from 5-fluorouracil-based chemotherapy as the standard backbone chemotherapy for GC in the first-line setting.

## 4. Conclusions

NO LIMIT is a single-arm phase 2 study evaluating the efficacy and safety of NIVO+IPI in the first-line setting for MSI-H GC, which accounts for ~5% of all GC cases. We believe that the results of this study could have a substantial impact on the treatment of GC.

## Figures and Tables

**Table 1:** Efficacy of pembrolizumab monotherapy versus chemotherapy as first-line treatment for microsatellite instability-high (MSI-H) colorectal cancer in the KEYNOTE-177 study [9].

Treatment	ORR (%)	PFS 12m (%)	PFS 24m (%)	mPFS (Months)	mOS (Months)
Pembrolizumab	43.8	55	48	16.5	NA
Chemotherapy	33.1	37	19	8.2	NA

Abbreviations: ORR, overall response rate; PFS 12m, progression-free survival rate at 12 months; PFS 24m, progression-free survival rate at 24 months; mPFS, median progression-free survival; mOS, median overall survival.

**Table 2:** Efficacy of pembrolizumab monotherapy versus chemotherapy as first-line treatment for MSI-H gastric cancer with a programmed cell death-ligand 1 (PD-L1) combined positive score (CPS) of ≥1 in the KEYNOTE-062 study [12].

Treatment	ORR (%)	PFS 12 m (%)	mPFS (Months)	OS 12m (%)	OS 24m (%)	mOS (Months)
Pembrolizumab	57.1	43	11.2	79	71	NR
Chemotherapy	36.8	28	6.6	47	26	8.5

Abbreviations: ORR, overall response rate; PFS 12m, mPFS, median progression-free survival; OS 12m, overall survival rate at 12 months; OS 24m, overall survival rate at 24 months; mOS, median overall survival.

**Table 3:** Efficacy of nivolumab plus ipilimumab (NIVO+IPI) as first-line treatment for MSI-H colorectal cancer in the CheckMate-142 study [14].

Treatment	ORR (%)	PFS 12m (%)	PFS 24m (%)	OS 12m (%)	OS 24m (%)
NIVO+IPI	69	77	74	83	79

Abbreviations: MSI-H, microsatellite instability-high; ORR, overall response rate; PFS 12m, progression-free survival rate at 12 months; mPFS, median progression-free survival; mOS, median overall survival; NA, not available; PD-L1, programmed cell death–ligand 1; CPS, combined positive score; OS 12m, overall survival rate at 12 months; NR, not reached.

**Table 4:** Eligibility criteria.

Key Inclusion Criteria	Key Exclusion Criteria
(1) Histologically confirmed gastric or esophagogastric junction adenocarcinoma. (2) Unresectable advanced, recurrent, or metastatic disease. (3) Confirmed MSI-H status by the MSI-IVD Kit (FALCO). However, subjects found to be MSI-H by other assays or to be mismatch repair (MMR)-deficient by immunohistochemistry are also eligible. In this case, MSI-H must be confirmed with the MSI-IVD Kit after enrollment. (4) Not amenable to curative approaches such as definitive chemoradiation or surgery. (5) No prior systemic anticancer treatment given as primary therapy for advanced or metastatic disease. (6) Prior adjuvant, neoadjuvant, or definitive chemotherapy, radiotherapy, or chemoradiotherapy for locally advanced gastric cancer is permitted if given as part of a curative-intent regimen and completed before enrollment. A recurrence-free period of 24 weeks is required after completion of neoadjuvant or adjuvant chemotherapy, or after completion of multimodal therapies (chemotherapy or chemoradiotherapy), for locally advanced disease. (7) ECOG performance status of 0 or 1. (8) At least one measurable lesion by computed tomography or magnetic resonance imaging per RECIST 1.1 criteria. (9) Tumor tissue must be provided for biomarker analysis in accordance with separately specified procedures.	(1) Failure to have recovered from major surgery or significant traumatic injury for at least 14 days before enrollment. (2) Radiation therapy for local control or pain control within 14 days prior to enrollment. (3) Blood transfusion or treatment with hematopoietics within 14 days before enrollment. (4) Other prior malignancy requiring active treatment within the 3 years before enrollment, with the exception of locally curable cancers that have been apparently cured, such as basal or squamous cell skin cancer, superficial bladder cancer, or carcinoma in situ of the prostate, cervix, or breast. (5) Active or suspected autoimmune disease. Type 1 diabetes mellitus, residual hypothyroidism due to autoimmune thyroiditis requiring only hormone replacement, and skin disorders (such as vitiligo, psoriasis, or alopecia) not requiring systemic treatment are permitted for enrollment. In case of uncertainty, it is recommended that a coordinating investigator be consulted prior to obtaining informed consent. (6) Conditions requiring systemic treatment with either corticosteroids (>10 mg daily prednisone equivalent) or other immunosuppressive medications within 14 days of enrollment. Inhaled or topical steroids, and adrenal replacement steroid doses of >10 mg daily prednisone equivalent, are permitted in the absence of active autoimmune disease. (7) Prior treatment with anti-PD-1, anti-PD-L1, anti-PD-L2, anti-CD137, or anti-CTLA-4, or with any other antibody or drug specifically targeting T cell costimulatory or checkpoint pathways.

Abbreviations: MSI-H, microsatellite instability-high; ECOG, Eastern Cooperative Oncology Group; RECIST, Response Evaluation Criteria in Solid Tumors; PD-1, programmed cell death-1; PD-L1, programmed cell death-ligand 1; CTLA-4, cytotoxic T lymphocyte-associated protein-4.

**Table 5:** Details of nivolumab–ipilimumab combination regimens administered in clinical trials.

Cancer Type	Phase/Study	Nivolumab	Ipilimumab	Any TRAE		Discontinuation Due to AE (Any Grade)
				Any Grade	Grade 3 or 4	
Malignant melanoma [29]	Phase 3, CheckMate-067	1 mg/kg, q3w × 4 → 3 mg/kg, q2w	3mg/kg, q3w × 4	99.7% *	68.7% *	36.4%
Renal cell carcinoma [30]	Phase 3, CheckMate-214	3 mg/kg, q3w × 4 → 3 mg/kg, q2w	1 mg/kg, q3w × 4	93%	46%	22%
MSI-H CRC [13]	Phase 2, CheckMate-142 (second line)	3 mg/kg, q3w × 4 → 3 mg/kg, q2w	1 mg/kg, q3w × 4	73%	32%	13%
Small cell lung cancer [31]	Phase 2, CheckMate-032	1 mg/kg, q3w × 4 → 3 mg/kg, q2w	1 mg/kg, q3w × 4		0%	0%
		1 mg/kg, q3w × 4 → 3 mg/kg, q2w	3 mg/kg, q3w × 4	79%	30%	11%
		3 mg/kg, q3w × 4 → 3 mg/kg, q2w	1 mg/kg, q3w × 4	74%	19%	7%
Gastric cancer [32]	Phase 2, CheckMate-032	1 mg/kg, q3w × 4 → 3 mg/kg, q2w	3 mg/kg, q3w × 4	84%	47%	20%
		3 mg/kg, q3w × 4 → 3 mg/kg, q2w	1 mg/kg, q3w × 4	75%	27%	13%
Non-small cell lung cancer [33]	Phase 3, CheckMate-227	3 mg/kg, q2w	1 mg/kg, q6w	76.7%	32.8%	18.1%
MSI-H CRC [14]	Phase 2, CheckMate-142 (first line)	3 mg/kg, q2w	1 mg/kg, q6w	80%	22%	13%

* All adverse events. Abbreviations: TRAE, treatment-related adverse event; AE, adverse event; MSI-H, microsatellite instability-high; CRC, colorectal cancer.

## Data Availability

Data sharing is not applicable to this article.
